# Investigation of aggregation induced emission in 4-hydroxy-3-methoxybenzaldehyde azine and polyazine towards application in (opto) electronics: synthesis, characterization, photophysical and electrical properties

**DOI:** 10.1080/15685551.2016.1231039

**Published:** 2016-10-05

**Authors:** Sengottuvelu Dineshkumar, Athianna Muthusamy

**Affiliations:** ^a^ PG and Research Department of Chemistry, Sri Ramakrishna Mission Vidyalaya College of Arts and Science, Coimbatore, India

**Keywords:** Aggregation induced emission, azine, polyazine, oxidative polymerization, dielectric, confocal microscope

## Abstract

An azine monomer 4-hydroxy-3-methoxybenzaldehyde azine was synthesized by refluxing with ethanolic solution of vanillin with hydrazine hydrate. It was then converted into polyazine by oxidative polymerization. The structure of azine and polyazine was characterized by FT-IR, UV–visible, ^1^H-NMR and ^13^C-NMR. Spectral results suggest the formation of polymer, through C–C and C–O–C coupling of the phenylene and oxyphenylene. The relationship between the structures and photophysical properties of azine and polyazine was studied. Both azine and polyazine show, aggregation induced emission with increase in concentration in DMSO solution. The single crystal structure of azine suggesting the various inter and intra molecular interactions rigidify the conformation and locked the intramolecular rotations of the phenyl rings in the molecule. The inhibition of intramolecular rotation, J- aggregation and increase of conjugation impart the fluorescence in aggregated state. Additionally, the electronic properties namely orbital energies and resulting energy gap calculated theoretically by density functional theory (DFT).

## 1. Introduction

Design and development of luminescent materials are of great interest because of their applications in the development of fluorescence sensing and light emitting diode devices.[1] Although many of them have been found to be highly emissive in dilute solutions, with unity fluorescence quantum yield.[2] For most of the practical applications, the luminescent materials have to be used in the solid state (e.g. as thin films), where the luminophores tend to form aggregates. But, the organic luminophore aggregations are responsible for partial or even complete quenching of their emissions.

This aggregation caused quenching (ACQ) [3] effect has restricted the scope of technological applications of the luminophoric molecules. Development of fluorescent probes without ACQ is the need for various biological applications.[4] To achieve this, it is necessary to develop luminophoric materials whose aggregates can emit more efficiently than the non aggregates in solution.

In this background two unusual phenomena exactly opposite to ACQ are aggregation induced emission (AIE) [5] and aggregation induced emission enhancement.[6] In the former, a nonemissive material is induced to emit by aggregation thus behaving exactly opposite to the conventional ACQ luminophores. In the later, light emission of a chromophoric material is enhanced by aggregate formation. The major contributing factors for AIE(E) properties are ascribed to restricted intramolecular rotation, molecule complanation and formation of H/J-aggregate in the aggregated state which can block the non radiative decay channels and leads to induced emission and emission enhancement.[7, 8] In 2001, W. Tang and co-workers discovered AIE in a non-luminescent silole molecule becomes, luminescent through aggregate by the introduction of non-solvent.[9] A similar phenomenon was observed by the researchers with aryl substituted pyrrole, salicyladehyde azine derivatives and triphenylethylene.[10] But only very few reports are available regarding the aggregate formation with increase in concentration.[11] Azine derivatives are reported for both non solvent aggregate and concentration induced aggregates. The AIE (E) characteristics have been a great potential for the development of distinct optical applications, from sensing to light emitting devices.[12] Besides, AIE azines are having fascinating physical and chemical properties, azines and their derivatives have been extensively used in dyes,[13] biological,[14] pharmaceutical,[15] nonlinear optical ﬂuorophores,[16, 17] design of liquid crystals,[18] as ligands for the synthesis of novel organometallic compounds [19] and other material applications.[20]

Polyazines (PAZs) are well known polymeric materials, its synthesis can be traced back to as early as 1891 when Curtius obtained the ﬁrst insoluble microcrystalline PAZ from the reaction between a diacetyl compound and an equimolar hydrazine hydrate.[21] PAZs exhibit interesting properties such as thermal,[22] mechanical stability,[23] semi conductivity [24] and non-linear response.[25] They are very promising candidate for electronic, optoelectronic and photonics.[26] PAZs do not undergo oxidative doping with iodine. Rather, these materials react with *I*_2_ to form iodonium complexes. PAZs contain a diamine linkage could be used to chelate transition metal ions. This could serve a dual purpose: to restrict rotations and to provide a method to influence the electronic structure of the polymer by varying the charge or bonding properties of the chelated metal complex. PAZs are potentially very suitable class of semiconducting materials for organic thin film transistors.[27]

Generally, polyazines are synthesized by the polycondensation reactions has some disadvantages, like special reaction conditions, high temperature and using special catalyst in some cases.[28] For these reasons oxidative polymerization (OP) of corresponding monomer is employed.[29] There are some major advantages of this method because the use of water medium make environmentally safe and also cheap and uncomplicated structured oxidant such as NaOCl, H_2_O_2_ and air O_2_.[30] This method allows high scale production as well as simple isolation and purification procedures.[31] Moreover, few papers dedicated to polyazines obtained by OP of various azine chromophores have been published and some of these demonstrate high photo efﬁciency.[32–34]

In this work, the synthesized 4-hydroxy-3-methoxybenzaldehyde azine was converted to polyazine via OP. The structural characterization of synthesized azine and polyazine was carried out by using FT-IR, ^1^H-NMR and ^13^C-NMR. AIE characteristic in azine and polyazine was examined by using UV–visible and fluorescence spectroscopy. We show that the molecular structure, conformational twisting, structural rigidification and packing pattern play major roles in the photophysical processes of azine, which provide a convenient way to have a better understanding on the AIE phenomenon.

## 2. Experimental

### 2.1. Materials

4-Hydroxy-3-methoxybenzaldehyde, Hydrazine hydrate, were purchased from Aldrich chemical company. NaOCl (6% aqueous solution) and all solvents were obtained from Merck Chemical Co. and used as supplied.

### 2.2. Synthesis of 4-hydroxy-3-methoxybenzaldehyde azine

**Scheme 1.**
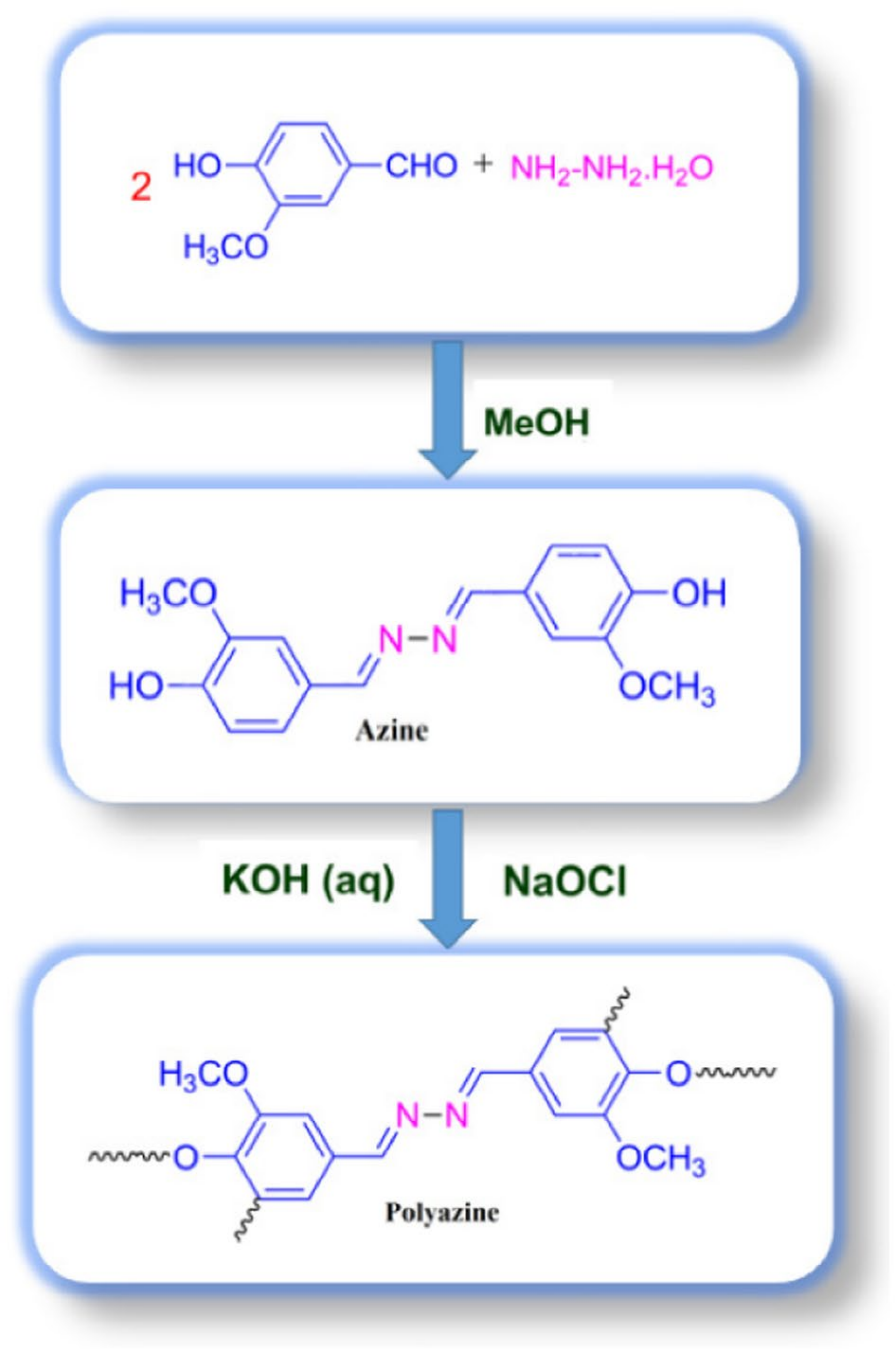
Synthesis of azine and polyazine.

An methanolic solution of 4-hydroxy-3-methoxybenzaldehyde (0.02 mol) was added into an methanolic solution of hydrazine hydrate (0.01 mol). On refluxing the mixture for one hour an yellow coloured solid was obtained (Scheme 1). The solid product obtained was filtered, washed with cold methanol and dried in vacuum oven at 60 °C. The elemental analysis of the azine data is in good agreement with the calculated values.

Melting point: 181 °C, Anal. calc. for HMBH: C; 63.99%, H; 5.37%, N; 9.33%. Found: C; 64.02%, H; 5.31%, N; 9.37%. ^1^H-NMR (DMSO-d_6_): *δ* ppm, 9.71 (s, 2H, –OH), 8.58 (s, 2H, –CH=N–), 7.46 (d, 2H, Ar-Hc), 7.26 (d, 2H, Ar-Hb), 6.88 (d, 2H, Ar-Ha), 3.84 (s, 2H, –OCH3). ^13^C-NMR (DMSO**-**d_6_): *δ* (ppm), 161.03 (C8-CH=N–), 150.35 (C1-ipso), 148.45 (C6-ipso), 126.01 (C4-H), 123.93 (C3-H), 115.98 (C2-H), 110.59 (C5-H), 56.02 (C7-OCH_3_).

### 2.3. Synthesis of poly(4-hydroxy-3-methoxybenzaldehyde azine)

Polymer was synthesized through OP of 4-hydroxy-3-methoxybenzaldehyde azine with NaOCl as an oxidant.[35] The monomer (0.003 mol) was dissolved in aqueous KOH (0.003 mol) in a 100 ml three-necked round bottom flask. After heating the mixture to 60 °C NaOCl was added drop wise over about 20 min. The reaction mixture was heated to 90 °C for 24 h (Scheme 1). The mixture was cooled to room temperature and neutralized with 0.003 mol of HCl solution. The brown coloured polyazine solid obtained was filtered, washed with hot water, methanol for separating mineral salts and unreacted azine, and then dried in a vacuum oven at 60 °C. ^1^H-NMR (DMSO): *δ* (ppm), 9.82 (s, 2H, terminal –OH), 8.64 (s, 2H, –CH=N–), 7.47 (d, 2H, Ar-Hc), 7.26 (d, 2H, Ar-Hb), 6.89 (d, 2H, terminal-Ha), 3.83 (s, 2H, –OCH_3_). ^13^C-NMR (DMSO): *δ* (ppm), 161.08 (C8-CH = N–), 150.68 (C1-ipso), 148.62 (C2-ipso), 126.49 (C4-H), 124.30 (C3-H), 116.01 (C2-H), 110.69 (C5-H), 56.04 (C7-OCH_3_).

### 2.4. Characterization

The FT-IR spectra were recorded in KBr pellets in the region 400–4000 cm^−1^ using Perkin Elmer FT-IR 8000 spectrophotometer. UV–visible spectra were recorded in DMSO solution with Systronics double beam UV–visible spectrophotometer 2202 in the range 200–800 nm. ^1^H-NMR and ^13^C-NMR spectra were recorded on Bruker AV400 MHZ spectrometer by using DMSO-d_6_ as a solvent. TG-DTA measurement was made in NETZSCH STA 409 PC thermal analysis equipment between 30 °C and 800 °C (in N_2_; rate, 10 °C/min). The molecular weight of polyazine was determined by gel permeation chromatography (GPC) using Polystyrene standard and eluted in Dimethylacetamide (DMAC) at a flow rate of 0.5 ml/min at 25 °C on a Water Alliance GPC model (GPC, Water 515 HPLC) fitted with water 2414 Refractive index detector and Styragel HMW 6E DMF Column. The surface morphology of azine and polyazine was monitored using JEOL Model JSM – 6390LV microscope.

### 2.5. Photophysical properties

The emission spectra of azine and polyazine were recorded on a Jobin Yvon Horiba Fluoromax-3 spectroﬂuorometer in DMSO solvent. Leica TCS SP2 model confocal microscopy was used to observe green colour emissions when excited at 440 and 420 nm respectively. Life time decay analysis of azine and polyazine was carried out by using time correlated single photon counting (TCSPC) technique with micro channel plate photomultiplier tube (Hamamatsu, R3809U) as the detector. The second harmonics (400 nm) output from the mode-locked femto second laser (Tsunami, spectra physics) was used as the excitation source. The instrument response function of TCSPC system is ~50 ps. The data analysis was carried out by the software provided by IBH (DAS-6), which is based on reconvolution technique using nonlinear least-squares methods.

### 2.6. Crystallographic analysis

X-ray diffraction of azine was collected on an Oxford Xcalibur Gemini EOS CCD diffractometer using Mo-Kα radiation. CrysAlisPro (Version 1.171.33.55) was used to perform cell refinement and data reduction. SHELX-TL was used to solve and refine the data. All non-hydrogen atoms were refined anisotropically by full-matrix least squares on the F2 using SHELXL program. The thermal ellipsoid plot and crystals packing diagrams were generated using the program ORTEP and MERCURY software package.

### 2.7. Theoretical studies

Quantum theoretical calculations were carried out using DFT at B3LYP/6-31(d) basis in the Gaussian 09 package. Based on the optimized geometries the energy and electronic distribution of molecular frontier orbitals were calculated in vacuum phase.[36]

### 2.8. Electrical properties

The electrical conductivity of the azine and polyazine was measured on a Keithley 6517B Electrometer using four point probe technique. Iodine doping was carried out by exposing azine and polyazine with iodine vapour at atmospheric pressure in a desiccator at room temperature. The dielectric measurements were carried out using two probe method with the help of LCR metre bridge (Hewlett Packard Model HP 4284) in the frequency range 50 Hz–5 MHz.

## 3. Results and discussion

The azine was polymerized by oxidative polycondensation by using NaOCl as an oxidant at 90 °C in aqueous alkaline medium. When the azine interacts with the oxidant NaOCl, it forms a brown coloured phenoxy radical. This phenoxy radical undergo resonance to form two types of stabilized radical structures (R1 and R2). These radicals undergo coupling to produce dimer, trimer, oligomer and finally polymer (Scheme 3).

The solubility of the azine and polyazine tests was done in different solvents by using the 1 mg sample in 1 ml of solvent at room temperature. The azine is soluble in acetone and ethanol, methanol, THF, DMSO, DMF, DMAC and insoluble in CHCl_3_, CCl_4_, DCM and benzene. The polyazine is soluble in DMF, DMSO, DMAC and sparingly soluble in THF, acetone and ethanol, methanol and insoluble in CHCl_3_, CCl_4_ and DCM and benzene.

Molecular weight determination of the polyazine was done by GPC at 30 °C with DMAC as an eluent at a flow rate of 0.5 ml/min using the refractive index detector. The number average molecular weight (Mn), weight average molecular weight (Mw) and PDI values were found to be 25,811 g/mol, 80,098 g/mol and 3.1 respectively. The high PDI value of polyazine is due to the two types of oxidative coupling C−C and C−O−C mechanism in polymerization, forms the polyazine as highly branched with structural heterogeneity.[37] The –OCH_3_ group in polyazine helps to dissolve it to a great extent, further the growing chain of polymer remains soluble in the medium for a long time and long polymer molecules are obtained.

### 3.1. Structural characterization

**Figure 1.**
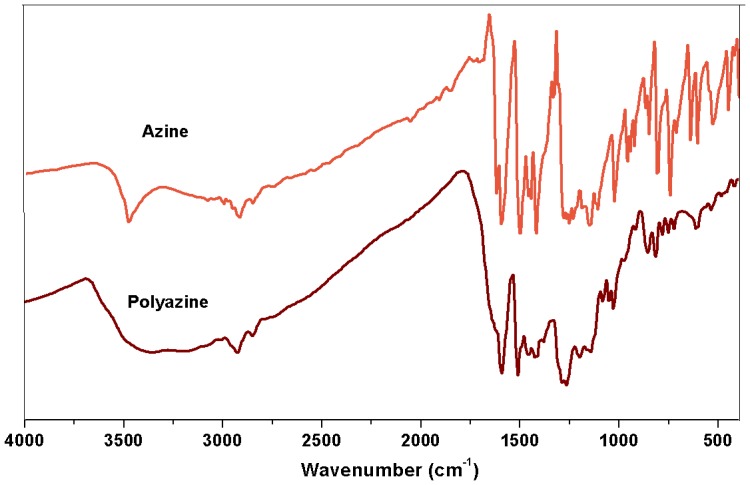
FT-IR spectra of azine and polyazine.

The FT-IR spectra of azine and polyazine are shown in Figure 1. The FT-IR spectrum of the monomer shows the stretching vibration bands of Ph–OH, Ar–H, –CH=N–, –N=N– and phenolic C–O appeared at 3479, 3082, 1600, 1423 and 1250 cm^−1^ respectively. It is observed that after polycondensation, due to extended conjugation of polymer, the peaks in FT-IR spectrum of polyazine becomes broadened. The peak at 3400 cm^−1^ is assigned to the stretching vibration of phenolic –OH and other peaks at 3008 cm^−1^, 1593, 1427 and 1270 cm^−1^ are attributed to the Ar–H, –CH=N–, –N=N– and phenolic–C–O groups respectively.[38] The decrease in intensity of the vibration band in the range of 3350–3500 cm^−1^ may be due to the participation some of the –OH groups in the polymerization reaction via C–O–C coupling. Moreover, the band at 1200 cm^−1^ corresponds to the oxyphenylene (C–O–C) linkage is an evidence for it. The spectrum of polyazine shows a significant decrease in the intensity of aromatic C–H vibrations (850–600 cm^−1^) when compared with its azine. This proves that the OP proceeded at the expense of aromatic hydrogen for C–C coupling.[39]

**Figure 2.**
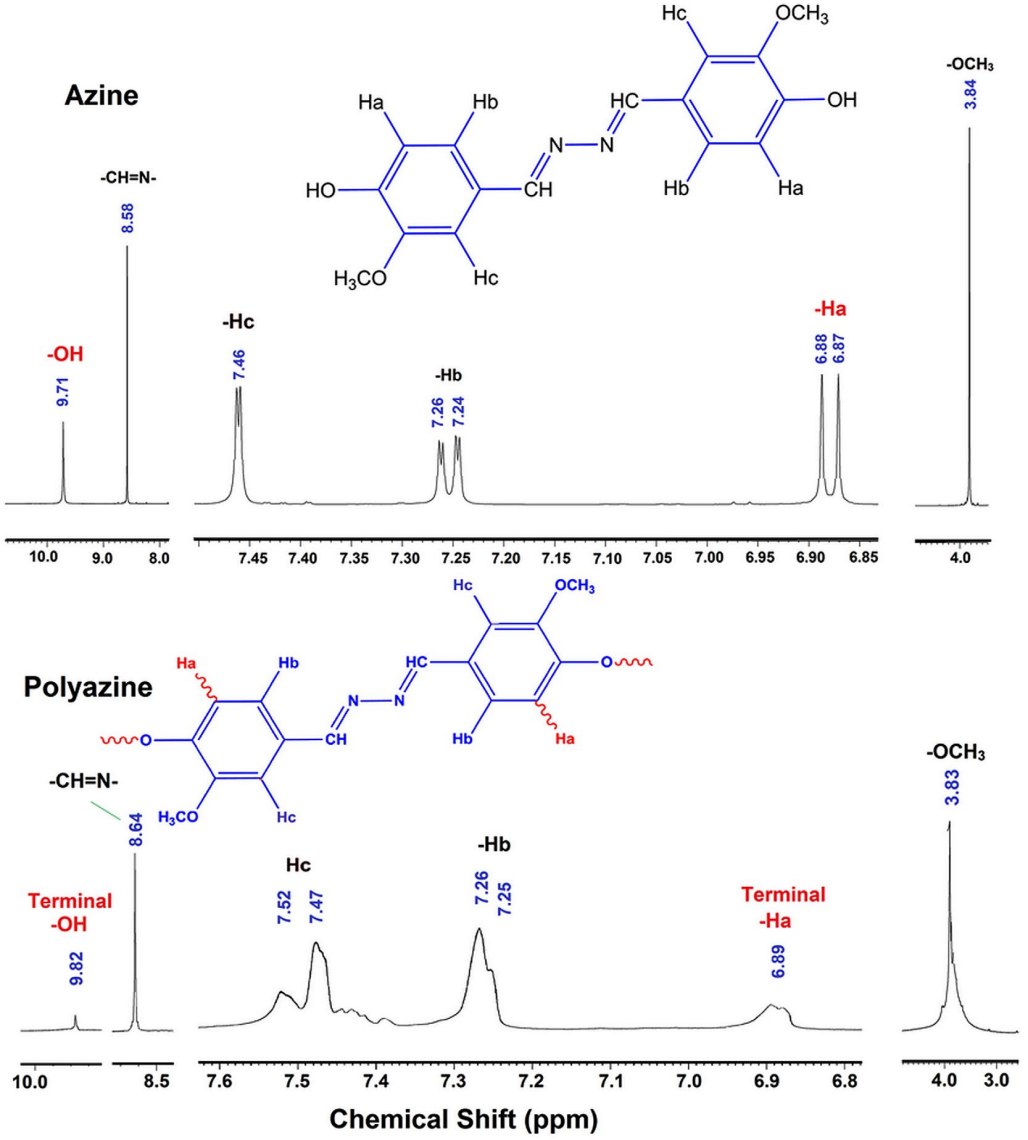
^1^H-NMR spectra of azine and polyazine.

The ^1^H-NMR spectra of azine and polyazine are shown in Figure 2. The sharp peaks of azine become broader and increased in number on polycondensation. The spectrum of azine shows phenolic OH, –CH=N– and –OCH_3_ as singlet at 9.71, 8.58 and 3.84 ppm respectively. The aromatic protons Ha and Hb appearing as doublet at 6.87 and 7.24 ppm respectively, whereas Hc proton is appearing as singlet at 7.46 ppm. The appearance of a single peak for various aromatic protons shows that the rings of azine are in same plane. It is further verified by crystallography and is discussed in the respective part. The ^1^H-NMR spectrum of polyazine does not show peaks of phenolic –OH and Ha significantly, which indicates the elimination of the Ha proton by C–C coupling and the elimination of –OH proton by C–O–C coupling. Kaya et al. have previously studied the types of radical forms and coupling selectivity of azomethine containing phenolic monomers by oxidative polycondensation.[40]

**Figure 3.**
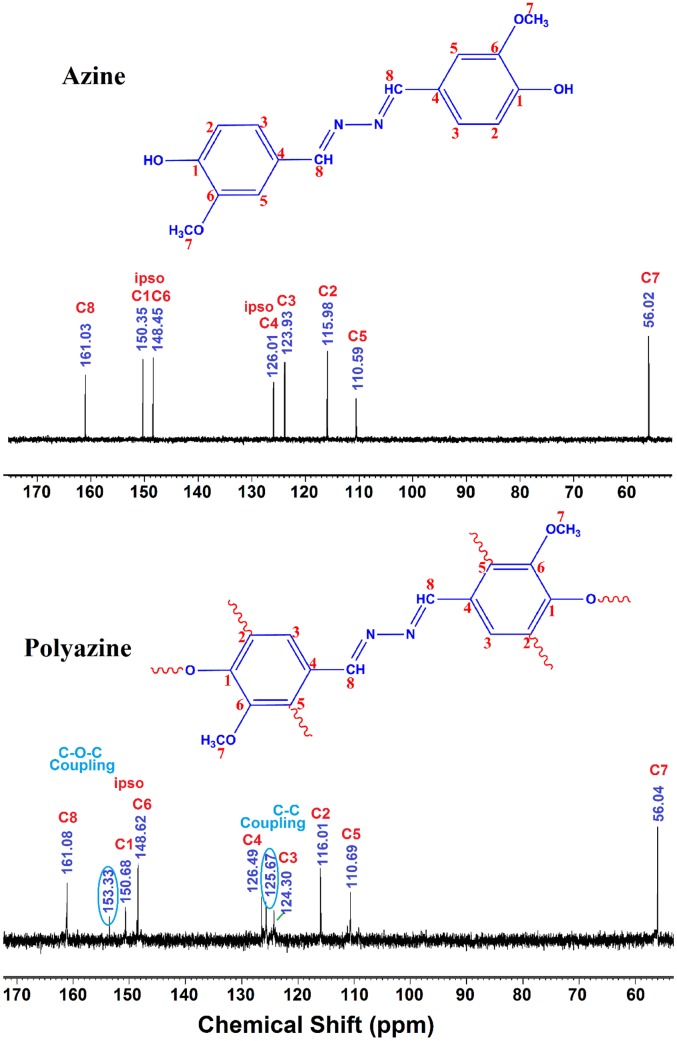
^13^C-NMR of azine and polyazine.

**Scheme 2.**
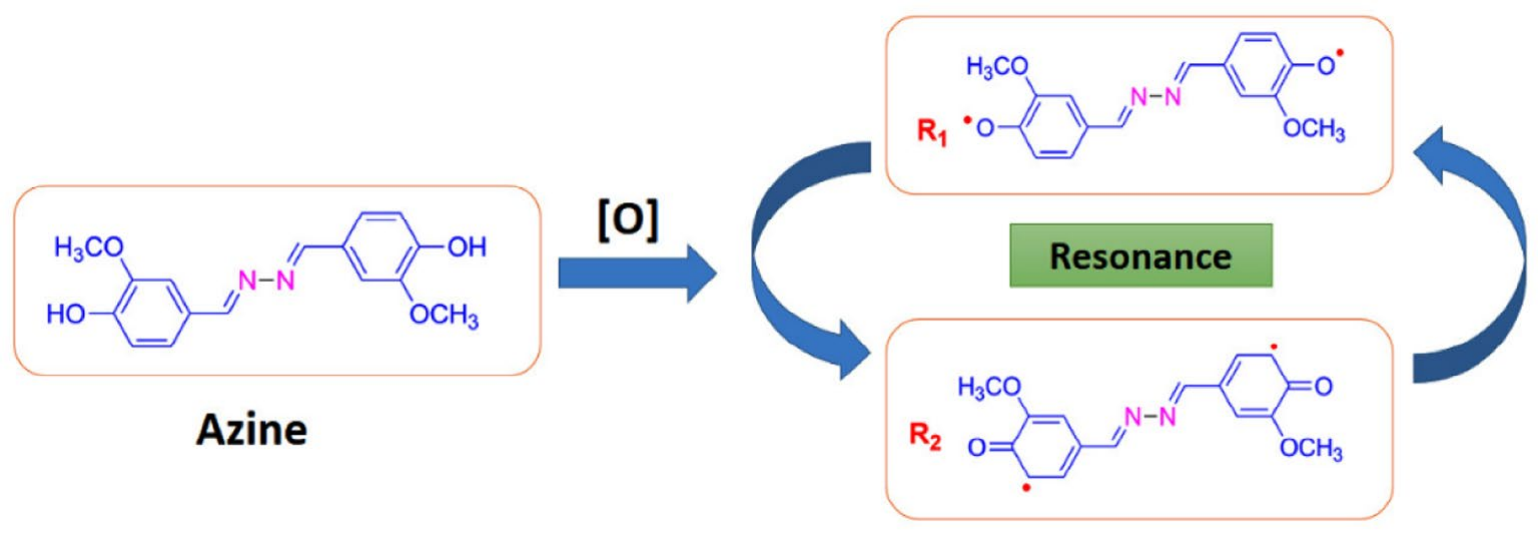
Possible radical types for azine.

**Scheme 3.**
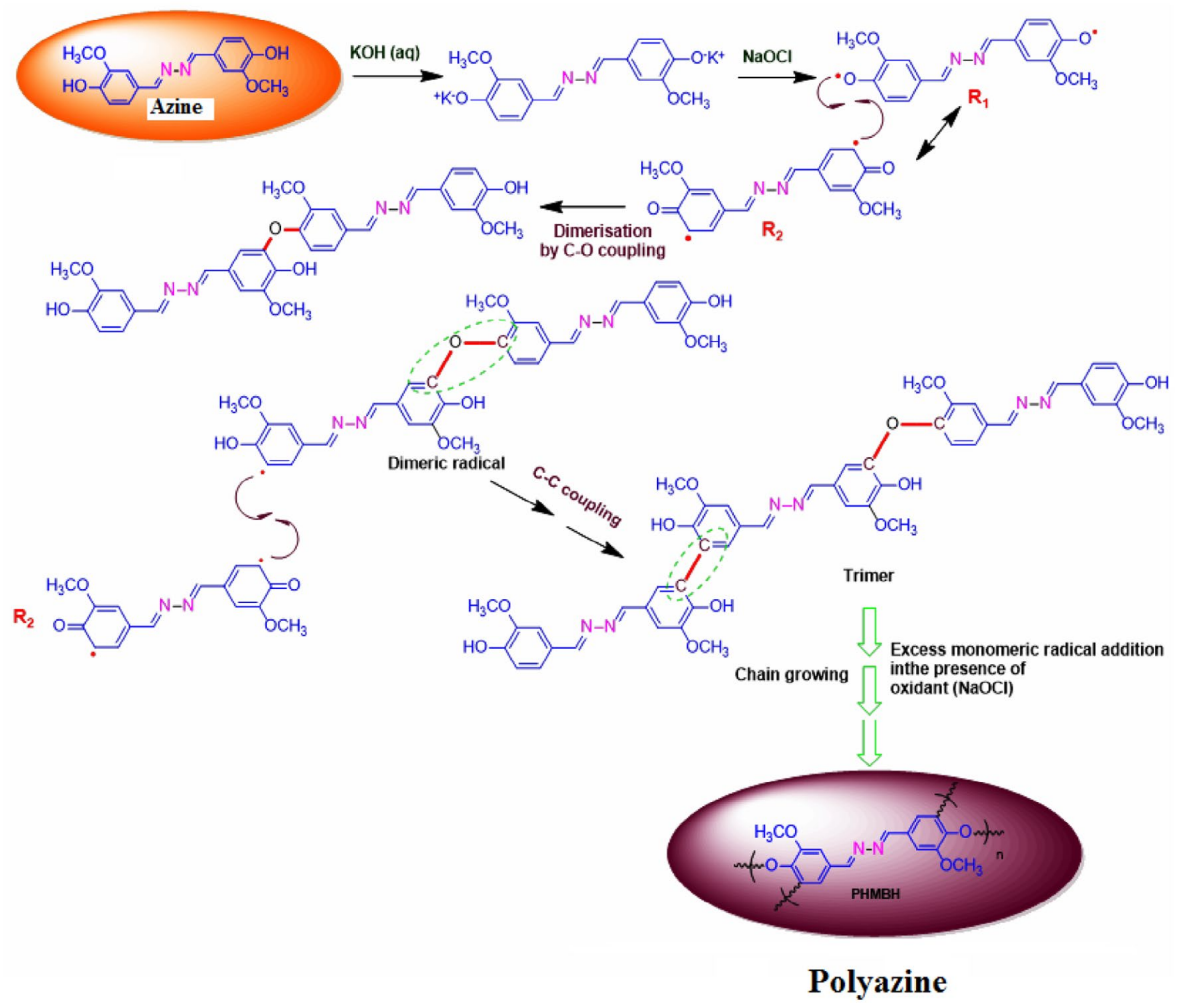
The reaction mechanism and combinations of phenylene (C–C) and oxyphenylene (C–O–C) units.

The ^13^C-NMR spectrum of polyazine (Figure 3) the signals of carbon is observed at 161 (C8-ipso), 150.35 (C1-ipso), 153.33 (C-O-C), 148.62 (C6), 126.49 (C4-ipso), 124.31 (C3), 115.88 (C2), 110.69 (C5) and 56.43 ppm (C7) respectively. The peaks of C1 and C2 are observed at 150.35 and 115.98 ppm in azine is slightly shifted to 150.68 and 116.01 ppm in polyazine indicate, the crosslinking in polymer structure mainly proceeded by the coupling of C2 carbon and C1 (phenoxy radicals) during OP of azine. Figure 3, showed new peaks at 125.67 and 153.33 ppm is due to C–C and C–O–C coupling respectively. The possible radicalic combinations of azine are exhibited in Scheme 2. The oxyphenylene are involved in the formation of free radicals leading to polyazine formation and they appeared to be primarily in bond formation.[41] Thus the phenyl rings in the polyazine appear to be linked primarily at the ortho position (*R*2) and oxyphenylene (*R*1). From the FT-IR, ^1^H-NMR and ^13^C-NMR spectral analysis results suggest that the polymerization of the monomer by OP taking place through C–C (phenylene) and C–O–C (oxyphenylene) type coupling (Scheme 3).

**Figure 4.**
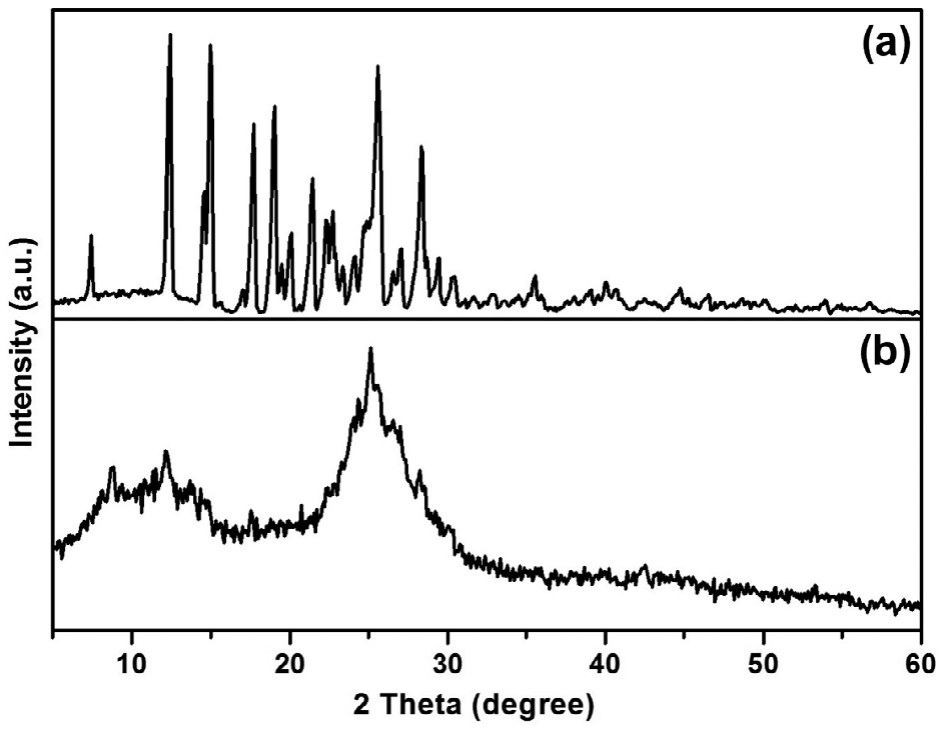
PXRD pattern of (a) azine and (b) polyazine.

**Figure 5.**
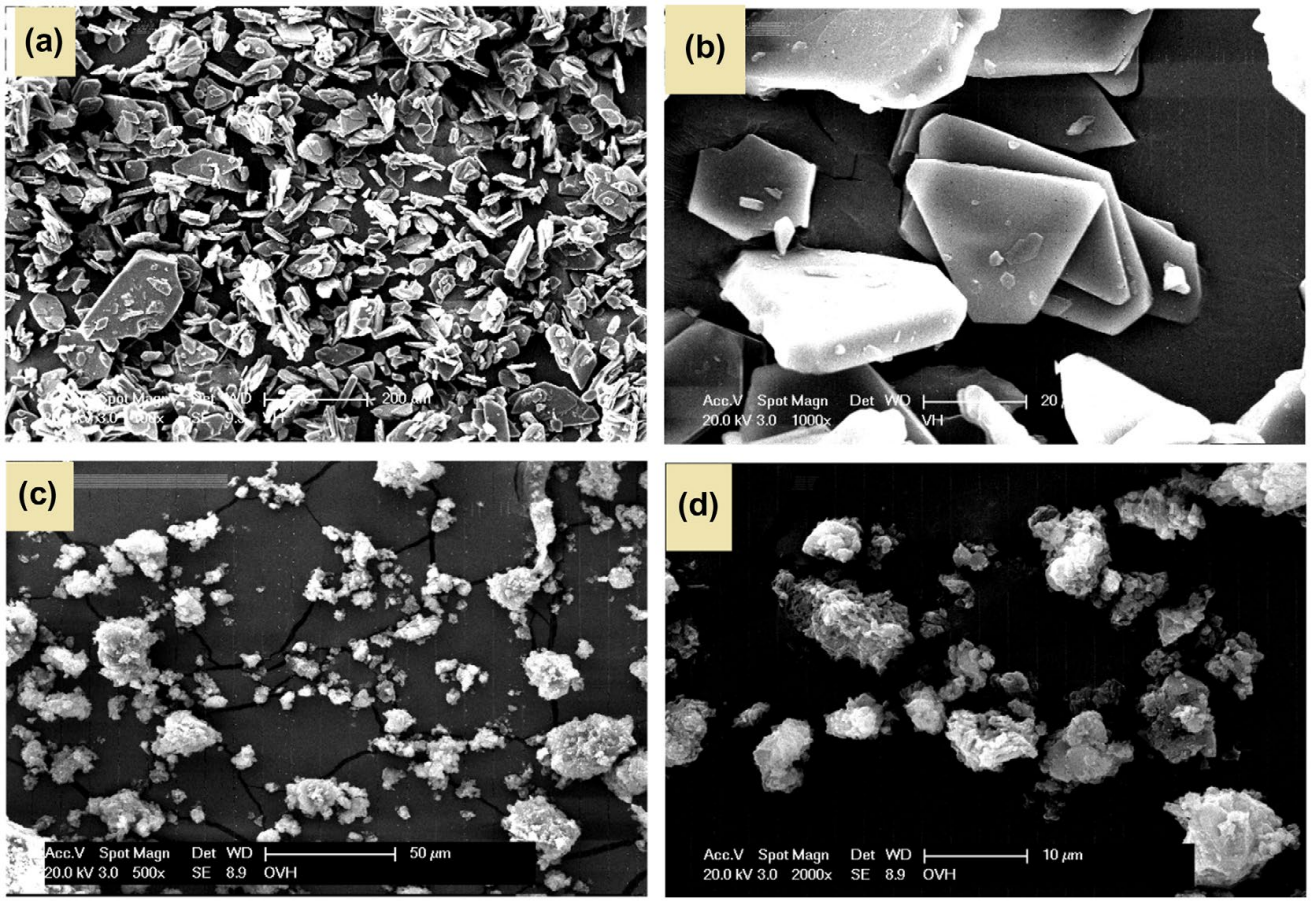
SEM photographs of (a and b) azine and (c and d) polyazine.

**Figure 6.**
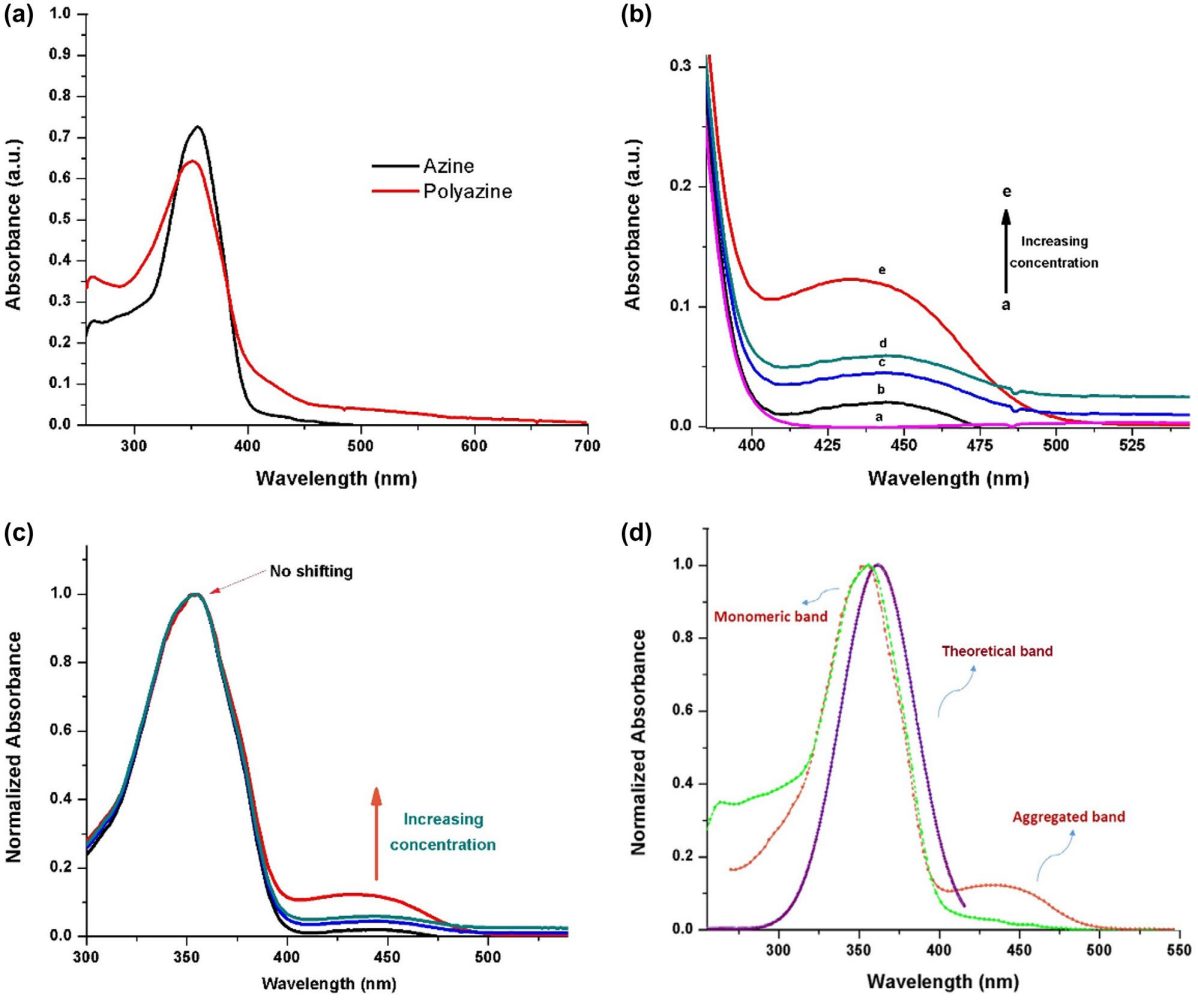
(a) Absorption spectra of azine and polyazine; (b) absorption spectra of azine at different concentration {3.3 × 10^−4^ M (i) to 2.2 × 10^−3^ M (v)}; (c) normalized absorption spectra of azine at maximum absorption wavelength as a function of concentration; (d) theoretical and experimental absorption spectra of azine.

**Figure 7.**
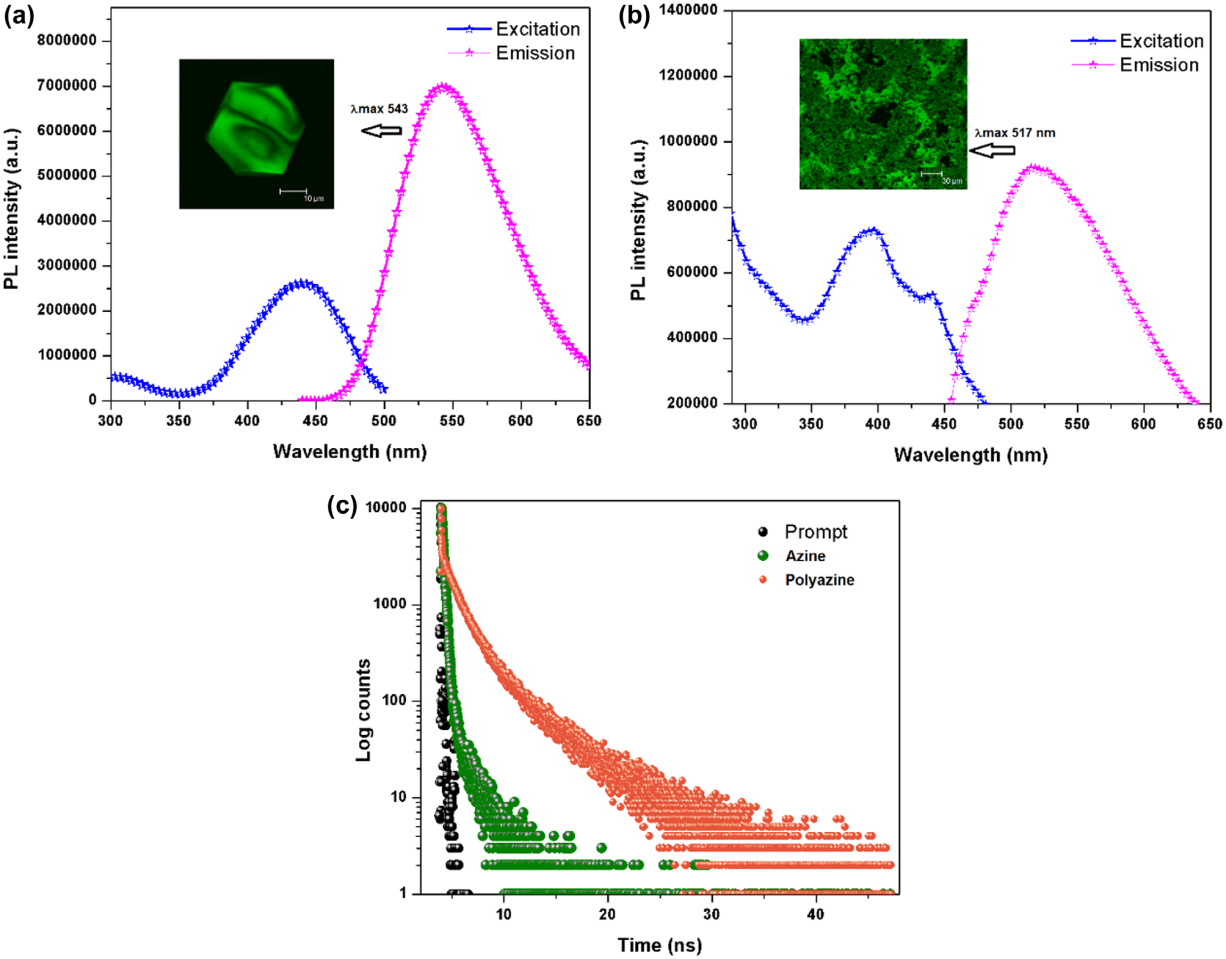
Fluorescence excitation and emission spectra of (a) azine and (b) polyazine; (c) fluorescence lifetime decay profile of azine and polyazine.

**Figure 8.**
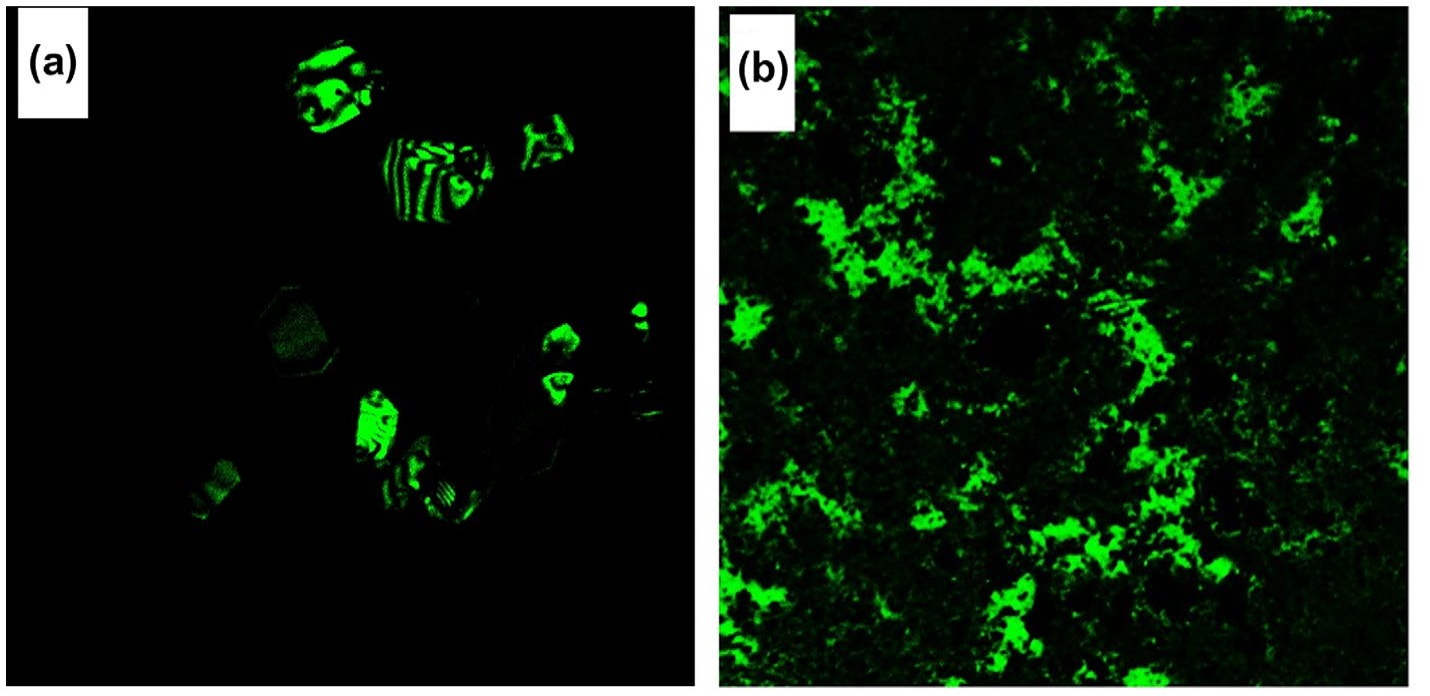
Confocal microscope images of (a) azine and (b) polyazine.

**Figure 9.**
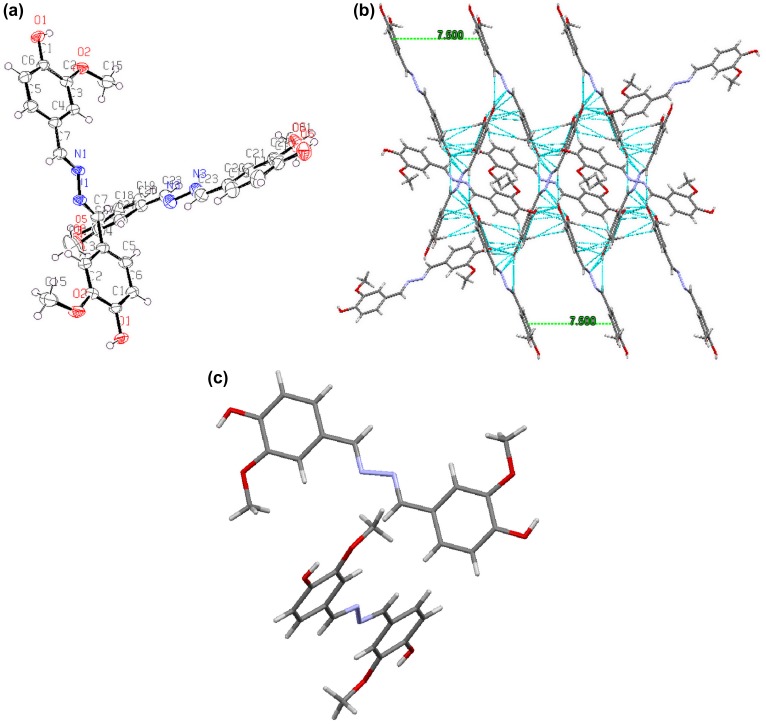
(a) ORTEP diagram of azine; (b) the centre to centre distances in azine π-dimers; (c) molecular structure of azine.

**Figure 10.**
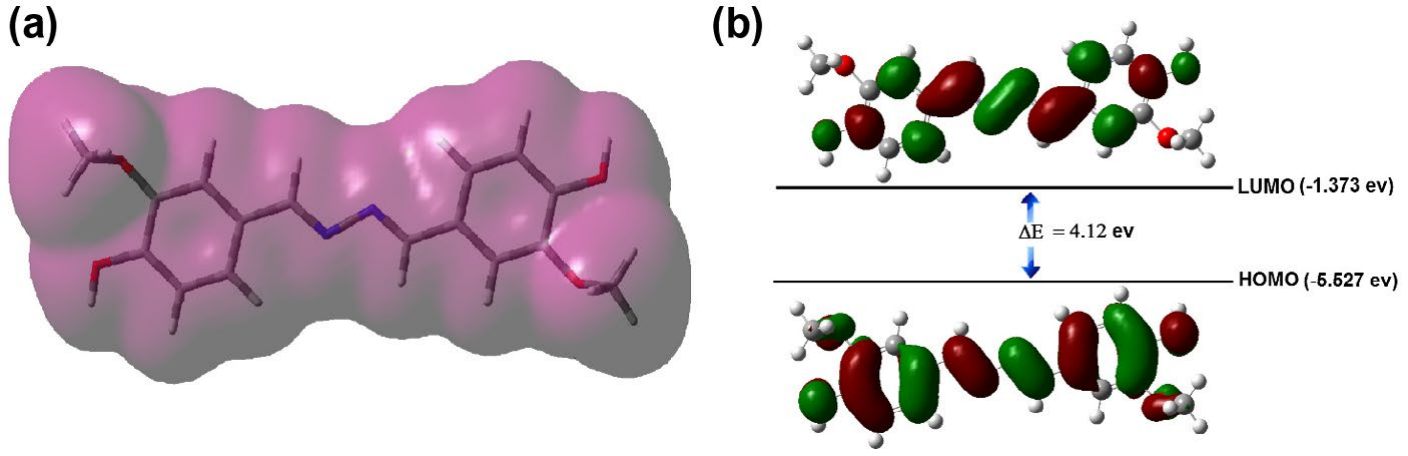
(a) Electron density distribution of synthesized azine; (b) HOMO and LUMO contours of azine molecule.

**Figure 11.**
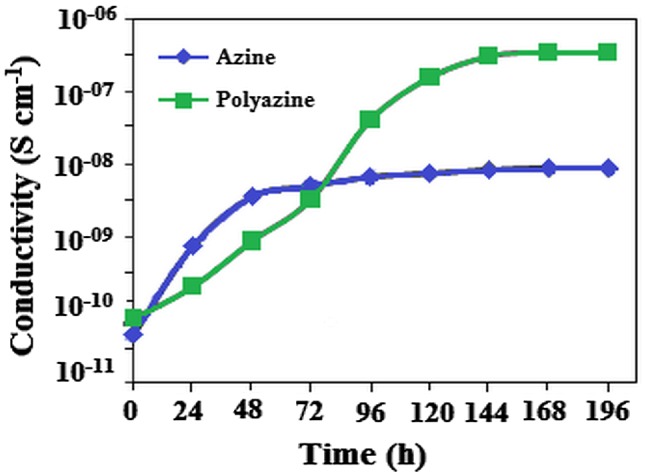
Electrical conductivity of iodine doped azine and polyazine vs. doping time at 30 °C.

**Figure 12.**
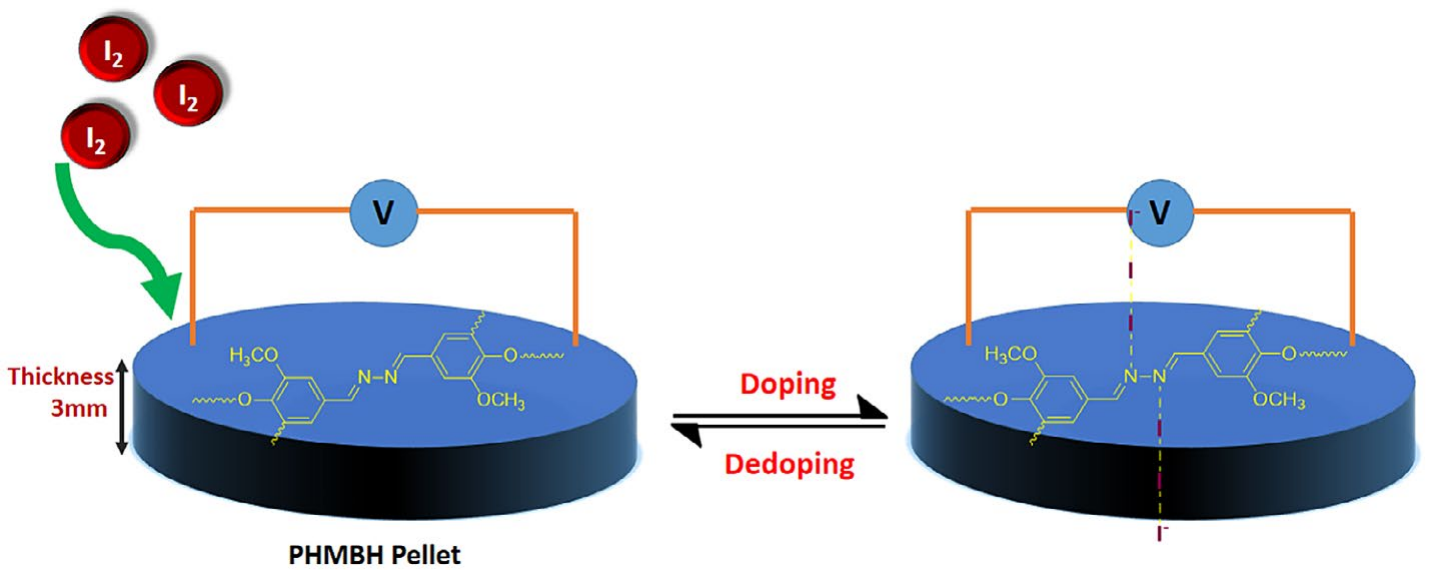
Coordination of iodine during polyazine doping.

**Figure 13.**
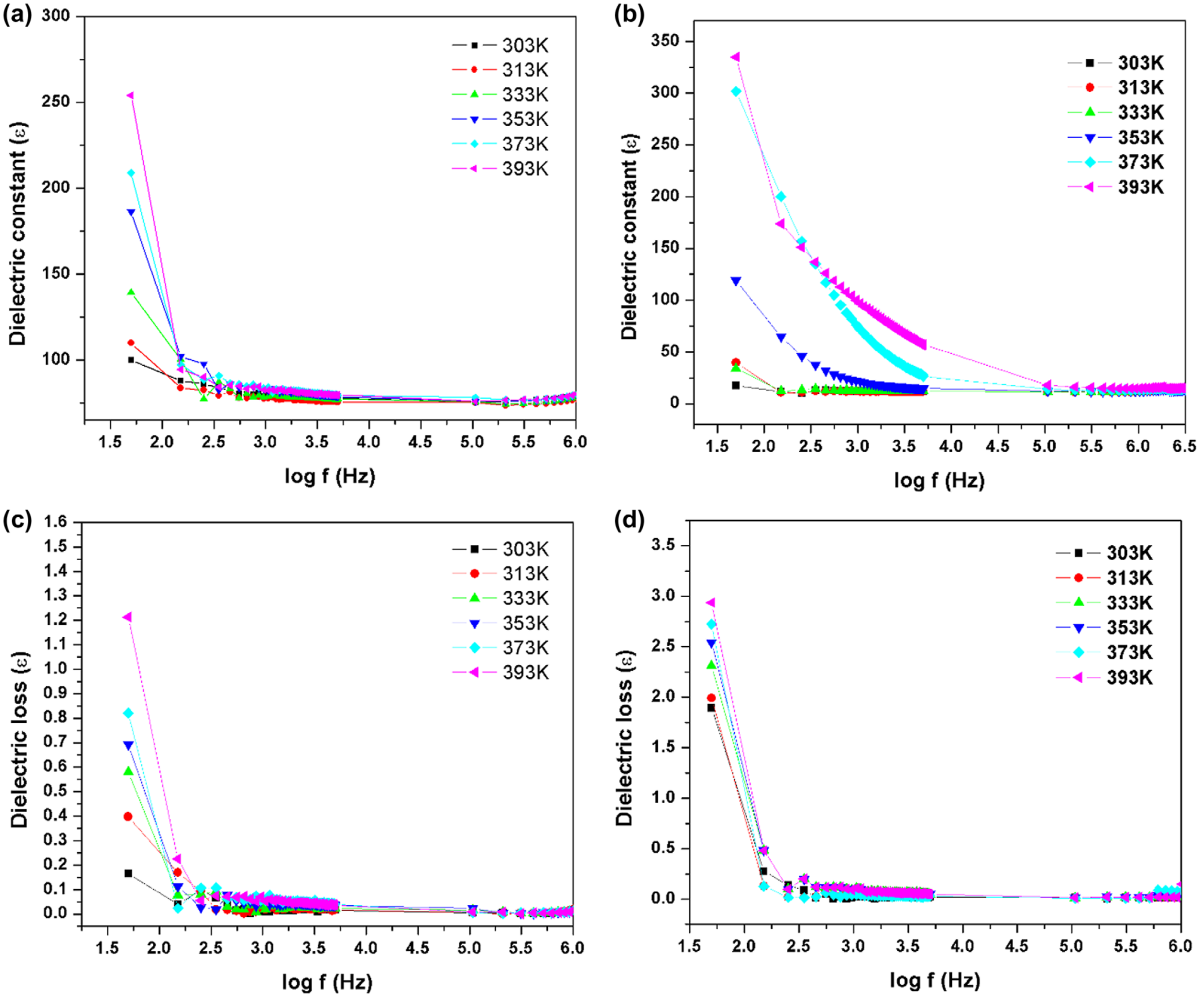
(a and b) Dielectric constant, (c and d) dielectric loss vs. frequency in different temperature.

**Figure 14.**
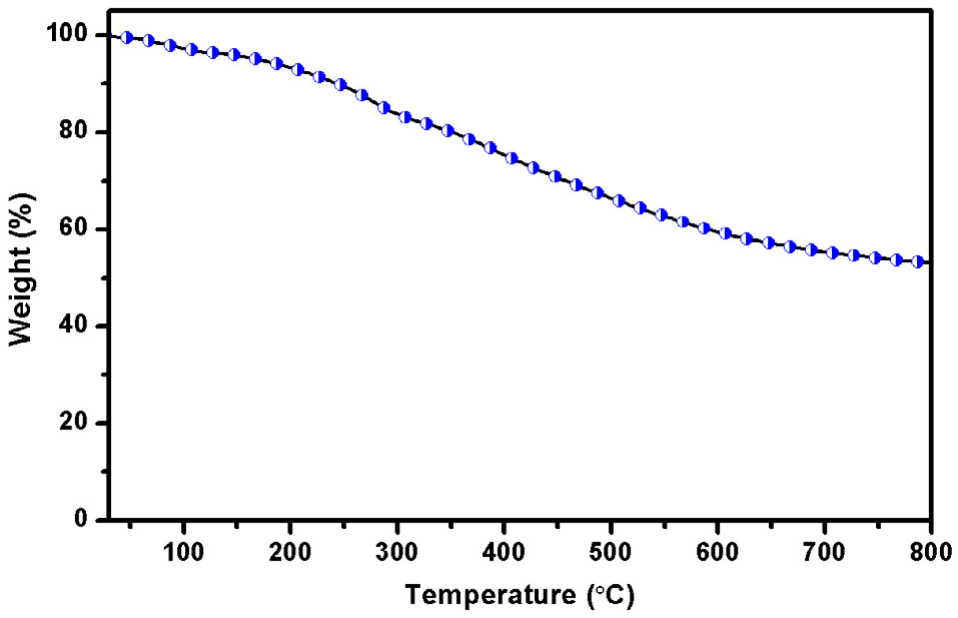
TGA traces of polyazine.

### 3.2. Morphological characterization

Powder X-ray diffraction pattern of azine and polyazine is shown in Figure 4. It shows that azine has a crystalline structure with sharp diffraction peaks, whereas the polyazine showed amorphous structure with broad diffraction peaks centred at 13 and 24 (2*θ*) indicating the of non-homogeneity arises out of the two types of linkages existing in polyazine. Moreover, the non-plane orientation of phenyl ring (as proved with ^1^H-NMR) and steric repulsion between the phenyl rings might disturb the formation of ideal crystalline structure.[42] The SEM micrographs also evident in morphology changes from azine to polyazine (Figure 5). The monomer showing crystalline plate like structure, but in the case of polyazine the particles are not wholly spherical but closer to granular shapes.

### 3.3. Photophysical properties

The photophysical properties of azine and polyazine were investigated by UV–visible, fluorescence spectroscopy and TCSPC) technique. UV–visible spectra of azine and polyazine (Figure 6(a)) exhibit a strong absorption band in the range of 300–400 nm corresponds to *π*–*π* ^*^ transition of the azine.[43] The molecular orbital pictures (HOMO–LUMO) of azine is displayed in Figure 10(b). The HOMOs and LUMOs are having *π* and *π* ^*^ character respectively. Therefore it concluded that the *S*_0_–*S*_1_ electronic transition is deﬁnitely *π*–*π* ^*^ in nature and it is allowed. The optical band gaps (*E*_g_) of azine and polyazine were calculated by using equation *E*_g_ = 1242/*λ*_onset_.[44] Where *λ*_onset_ is the onset wavelength which can be determined by the intersection of two tangents on the absorption edges. The optical band gap values for the azine and polyazine are 3.08 and 2.61 eV respectively. The decrease in optical band gap value of polyazine when compared with azine may be due to extended π-electron conjugation.[45]

In the absorption spectra of azine (Figure 6(a)) a level-off tail in the visible region is observed. Normally, the azine compounds are forming aggregates in certain solvents that could be the reason for this level-off tail appearance. To ascertain the formation of aggregates the absorption spectra was recorded at different azine concentrations in DMSO. The change in absorbance with concentration is shown in Figure 6(b). The spectra exhibited the usual strong absorption band at 354 nm with a weak band at 440 nm. The concentration dependence of azine resulted an aggregate band (bathochromic J-band), which is red shifted with respect to the non aggregated monomeric form and this aggregates is called as J-type aggregates.[46, 47] The analysis of the results suggested that if the concentration of azine is less than 6.6 × 10^−4^ M the aggregate will not be formed in the solution. But increasing azine concentration led to an increase of intensity of the monomeric band at 354 nm with the formation of an aggregate band at 440 nm. The development of the aggregate band at a concentration above 3.3 × 10^−4^ M at higher wavelengths suggest head to tail aggregate (J-band).[46, 47] The intermolecular forces such as dipole-dipole, van der walls may be responding to this kind of aggregation.[48]

Figure 6(c), shows the normalized absorption spectra of azine concentrations from 3.3 × 10^−4^ to 2.2 × 10^−3^ M in DMSO solution. The presence of aggregate was revealed by the appearance of a band at 440 nm. The monomer-dimer model suggested by Exciton theory is not enough to explain the findings and therefore, it is necessary to propose the formation of higher aggregates. The aggregation is formed by the association of azine molecules in which molecules were arranged one above the other. The normalized spectral curves of the azine (Figure 6(d)) revealed that the monomeric band at the different concentrations are overlaid. As we want to ascertain the assignments of experimental UV–visible spectrum theoretically, using DFT calculations we have obtained the maximum absorption of the azine. The theoretical UV absorption maximum is very much closer to the experimental value and is assigned to non aggregated form of azine.

Figure 6(d), shows the simulated excited state (*λ* Es) and the experimental visible absorption band (*λ* Ex) wavelengths. It can be observed that the experimental and the theoretical monomeric band are very close, but are far from the aggregated band of the azine. So we concluded from the experimental and theoretical results the absorption maximum at 354 nm corresponds to monomeric form and weak band at 440 nm corresponds to aggregate form of azine.

The ﬂuorescence characteristics of azine and polyazine were investigated in DMSO solution (Figure 7(a) and (b)). The AIE active azine was excited at 360 nm corresponds to the non-aggregate monomeric absorption, no emission was observed, whereas when the azine was excited on aggregation band maxima at 440 nm resulted a green emission. The experimental, theoretical absorption and crystallographic results clearly indicate that azine is AIE active. The polyazine in DMSO also exhibited a green emission with high intensities, when excited at 420 nm. The green colour emission of azine and polyazine was visualized using a confocal microscope by exciting the azine/polyazine solution coated dried glass sheet at 440 and 420 nm respectively. The confocal images of corresponding azine and polyazine are shown in Figure 8.

Life time decay analysis was carried out on the green emitting azine and polyazine using a TCSPC technique, with micro channel plate photomultiplier tube (Hamamatsu, R309U) as a detector and femto second laser as an excitation source, on excitation at 440 and 420 nm for azine and polyazine. Figure 7(c) shows the measured fluorescence transients obtained for the azine and polyazine. The fluorescence decay fit as multi exponential with life time *τ*_1_, *τ*_2_ and *τ*_3_. The life time decay of the azine and polyazine are shown in Table 1. The fluorescence decay (Figure 7(c)) of polyazine shows a longer relaxation time *τ*_2_ and *τ*_3_ for the second and third exponential and also shorter relaxation time *τ*_1_ for the first exponential. From the results, the decay constant determined for polyazine is longer than that of azine due to the extended *π*-electron conjugation in the polymer backbone. While comparing the fluorescence of monomer and polymer, polymer is showing less fluorescence intensity than the monomer. This is attributed to disrupted conformational structure of high molecular weight, bulky structured polymer.[49]

**Table 1.** Photophysical data of azine and polyazine.

Compound	*λ*_abs_ max (nm)	*λ*_em_ max (nm)	Stokes^a^ shifts (nm)	Life time (*τ*)				Optical^b^ band gap (eV)
				*τ*_1_ (ps)	*τ*_2_ (ns)	*τ*_3_ (ns)	*χ*^2^	
Azine	355, 430	543	113	195 (92.87%)	1.13 (7.02%)	–	1.32	3.08
Polyazine	352, 410	517	107	204 (4.78%)	1.40 (58.73%)	4.73 (36.49%)	1.07	2.61

^a^ From *λ*_max_ excitation (aggregation peak) and emission.

^b^ From *λ*_max_ onset of *π*–*π* ^*^ transition.

### 3.4. Mechanisms of induced emission

A good relationship is expected between the photophysical properties and molecular packing. So, to confirm that crystal of azine was prepared by slow evaporation from azine solution of MeOH/THF mixture. The crystal data and collection parameters are summarized in Table 2. Azine was crystallized in a monoclinic form with space group *P*2_1_/*c*. The ORTEP diagram of azine with the atom numbering scheme and some of the interactions in the azine molecule is depicted in Figure 9(a) and (b). It can be seen that two azine units are existing together in a unit cell. As shown in Figure 9(a), the most significant structural feature of azine was the presence of dislocation parallel *π*-dimers (Figure 9(c)) also known as head to tail *π*-dimers, which was favoured of the formation of J-type aggregation.[50] Figure 9(b), demonstrated various hydrogen bonds existed in the aggregated structure of azine. The flexible molecule of azine was restricted by various inter and intra molecular interaction, such as O–H⋯O (*d* = 2.973 Å), O–H⋯N (*d* = 2.818 Å) and O–H⋯O (*d* = 2.659 Å) between the adjacent molecules.

**Table 2.** Crystal and structure refinement data of azine.

Emprical formula	C_16_H_16_N_2_O_4_
Molecular weight	300.31
Crystal system	Monoclinic
Space group	*P*2_1_/*c*
*Z*	4
*D*	1.376 Mg m^−3^
*F*(0 0 0)	632
Crystal size	0.50 × 0.10 × 0.09 mm
*a*	11.62 (4) Å
*b*	7.5000 (8) Å
*c*	16.677 (9) Å
*β*	94.05 (15)°
*V*	1450 (5) Å^3^
*T*emperature	293 K
Mo *K*α radiation, *λ*	0.71073 Å
*R*[*F*^2^ > 2s(*F*^2^)]	0.045
*wR*(*F*^2^)	0.143
Measured reflections	5759
Independent reflections	2967
Reflections/restraint/parameters	2967/0/201
*θ* range	3.0–28.6°
	*h* = −14→14
	*k* = −9→6
	*l* = −20→20

Since the phenyl rings are slipped along the axis, there is no overlapping between the phenyl rings of the adjacent azine units, the C⋯C distance between the units remains as 5.00 Å, which is larger than the *π*–*π* stacking interaction distances found in other systems. Absence of the overlap between the units caused weakening of *π*–*π* interactions and the emission cannot be quenched. Therefore, the dipole–dipole interactions are the main driving forces for dimer formation. These strong intermolecular interactions direct the azine molecules to pack in a dislocated parallel manner with interruption of all aromatic *π*–*π* segments, which leads to arrest the intramolecular rotations of the phenyl rings and rigidify the conformation.[48] We conclude that the J-aggregation, inhibition of intramolecular rotation and higher conjugation led to induce the emission in the aggregated state.

### 3.5. Electronic structure

We have performed the quantum theoretical calculations using DFT, at B3LYP/6-31(d) basis in the Gaussian 09 package. Based on the optimized geometries the energy and electronic distribution of molecular frontier orbitals were calculated in vacuum phase. In azine molecule, the highest occupied molecular orbital (HOMO) is concentrated in the aromatic rings and in its neighbourhood (–CH=N–), indicating that the oxidation process should happen primarily in this region. This corresponds to the calculations of electron density, which indicate that this region has the highest density of negative charge (Figure 10). Lowest unoccupied molecular orbitals are localized on nonbonding Pz orbitals excluding double bonds and phenyl rings of the molecule. The simulation results including the optimized molecular configuration, energy levels and electron distribution of the HOMO and LUMO of azine are shown in Figure 10(b). The HOMO electrons are averagely distributed on the whole molecule’s backbone, but LUMO electrons are mainly in the centre azine bridge, which indicates certain intramolecular charge transfer tendency from HOMO to LUMO.[51] The HOMO and LUMO of azine were dominated by the orbitals from rotatable core and the phenyl units, which revealed that their absorption and emission derived from the *π*–*π* ^*^ transitions. The calculated energy levels of HOMO and LUMO and the corresponding energy gap are −5.527, −1.373 and 4.12 eV respectively. The theoretically calculated values are slightly far from the experimental values. The differences may be related to various effects of conformations of azine in vacuum, solid and solutions. Additionally, the comparison of calculated and experimentally measured UV–visible spectra (Figure 6(d)) of azine show good agreement, the difference between calculated and experimental *λ*_max_ value is less than 15 nm.

### 3.6. Electrical properties

#### 3.6.1. Electrical conductivity

Electrical conductivities of the azine, polyazine and change in conductivity with iodine doping time are determined. The electrical conductivities of the undoped azine and polyazine are 4.68 × 10^−11^ and 7.68 × 10^−11^ Scm^−1^ respectively. Figure 11, shows that the conductivity of the azine and polyazine with iodine doping at various intervals. When azine and polyazine on doping with iodine, it was found that the conductivity first increase greatly with doping time, and then tends to level-off. The increasing conductivity could indicate that a charge transfer complex between azine moiety and dopant iodine continuously formed. Therefore, the Figure 11 shows not only doping time/conductivity time relationship, but also indicates how the doping mechanism takes place. Our experimental results showed that a longer doping time is needed to obtain maximal (or saturated) conductivity.

The maximal or saturated conductivity values of azine and polyazine are found to be 8.83 × 10^−9^ and 3.44 × 10^−7^ Scm^−1^. The conductivity of polyazine increases with iodine doping time, which makes them as suitable candidate for gas sensing applications. Diaz et al. had suggested the conductive mechanism of iodine doped –CH=N– containing polymers.[52] According to the proposed mechanism iodine being an electro acceptor vapour could accept the non-shared electron pair of the nitrogen that results in forming polaron structure.[53] Coordination of iodine on the nitrogen atom of polyazine is shown in Figure 12. The coordination of iodine with –CH=N-polymers had been suggested in the literature.[54–56] The conductivity studies confirm that the synthesized polyazine can be used as semi-conductive materials in electronic, opto-electronic and photovoltaic applications.

#### 3.6.2. Dielectric studies

Dielectric studies for the azine and polyazine were carried out in the frequency range from 50 Hz to 5 MHz using Hioki LCR metre (353,250). The sample was placed between two copper electrodes which act as a parallel plate capacitor. Typical sample thickness was in the range of 2.5 mm. The variation of dielectric constant with log frequency is shown in Figure 13(a) and (b). The dielectric constant of the azine and polyazine were calculated by the given formula

$$\varepsilon_{r}=Cd/\varepsilon_{0}A$$

where *C* is the capacitance of the crystal, *d* is the thickness of the azine and polyazine, *ɛ* is the free space permittivity and *A* is the cross sectional area of the sample. The dielectric constant of both azine and polyazine are very low at high frequency and decreases slowly at higher frequencies and ﬁnally attains a constant value at very high frequencies (above 1 MHz). Dielectric constant of polyazine is increased at low frequency due to the intermolecular forces between polymer chains is minimized which enhances thermal agitation. At low temperature, the segmental motion of the chain is practically frozen and this will reduce the dielectric constant. At sufficiently high temperature, the dielectric constant is increased due to strong thermal motion which enhance the orientation of the dipoles. The high value of dielectric constant may be due to the four polarizations namely, space charge, orientation, electronic and ionic polarization and its low value at higher frequencies may be due to the loss of signiﬁcance of these polarizations gradually. The space charge polarization will depend on the purity and perfection of the materials.[57]

The variation of dielectric loss in log frequency is shown in Figure 13(c) and (d). From the plot, it is observed that the value of dielectric loss decreases with increase in frequency. The value of dielectric loss is high at lower frequencies and it is low at higher frequency regions. The characteristic of the low value of dielectric loss in the high frequency region suggests that the azine and polyazine are suitable for electro-optical device applications.[58] Very low dielectric loss material is used for radio frequency applications to abstain signal losses, but higher dielectric loss material can be tolerated for energy storage applications.

### 3.7. Thermal properties

TGA curves of the polyazine were given in Figure 14. The results indicated that the decomposition of the polyazine proceeds in one step. The weight loss of polyazine in the 30–100 °C is due to the occluded water molecules in the structure. After the removal of these water molecules, the weight loss of polyazine began at 205 °C. It indicates that the polymerization proceeds predominantly via frail C–O–C type coupling. This behaviour is derived from the conjugated structure of the main chain. The higher resistance (high char yield 53.26% at 800 °C) against high temperature of polyazine may be due to the long conjugated bond systems and higher aromatic content in the polymer skeleton. From TGA results show polyazine is more stable through temperature and thermal decomposition. The thermal behaviour has a critical influence on both the stability and the lifetime of luminescent devices.[59] It is concluded that this stable material can be used in LED applications.[60]

## 4. Conclusions

We demonstrated the synthesis, structural characterization, electrical and photophysical properties of azine and its polyazine. A non-fluorescent azine turned into fluorescent one due to aggregated induction of molecules with increase in concentration. Crystal structure analysis of azine revealed that dimers induced by long range order arrangement (J-aggregation), dipole-dipole interaction and inter molecular hydrogen bonds are responsible for induced emission, which restricted the intramolecular rotations and blocking the nonradiative processes. These AIE active compounds are suitable candidates for fluorescence sensing and light emitting diode applications. The optical band gap of the polyazine are lower than that of the azine. The theoretically calculated orbital values and energy gap are slightly greater than the experimental values. The differences may be related to various effects of conformations of azine in a vacuum, solid and solutions. Additionally, the calculated and experimentally measured UV–visible spectrum of azine are fairly close to one another. Thermal analysis results of polyazine have demonstrated that it has high resistance against thermal degradation. The carbine residue of polyazine at 800 °C is 53.26%. The polyazine has higher initial and maximal electrical conductivities than the monomer in doped and undoped forms. Azine and polyazine shows high dielectric constant value at low frequencies and these kind of materials are used as polarizable media for capacitors.

## Supporting information

Crystallographic data reported in this manuscript were deposited with Cambridge Crystallographic Data Centre as supplementary publication No. CCDC-1453375. These data can be obtained free of charge via http://www.ccdc.cam.ac.uk/conts/retrieving.html, or email: deposit@ccdc.cam.ac.uk.

## Supplemental data

Supplemental data for this article can be accessed here [http://dx.doi.org/10.1080/15685551.2016.1231039].

## Disclosure statement

No potential conflict of interest was reported by the authors.

## Supplementary Material

Supplementary_data.docx
