# Supplementary material for: Investigation of aggregation induced emission in 4-hydroxy-3-methoxybenzaldehyde azine and polyazine towards application in (opto) electronics: synthesis, characterization, photophysical and electrical properties
Source: Des Monomers Polym. 2016 Oct 5;20(1):234–49. doi: 10.1080/15685551.2016.1231039 (PMC5812189; doi:10.1080/15685551.2016.1231039)
Supplement: Supplementary_data.docx [file TDMP_A_1231039_SM4314.docx]

**Table 1 Crystal Data and Refinement Details**

Table 1. Inter and Intra molecular Hydrogen Bonding

| **Compound** | **D-H…A** | **D-H** | **H…A** | **D…A** | **D-H…A** |
| --- | --- | --- | --- | --- | --- |
| Azine | O1-H1...O2 | 0.82 | 2.21 | 2.659(9) | 115 |
|  | O1-H1...N1 | 0.82 | 2.08 | 2.818(10) | 149 |
|  | O5-H5A...O6 | 0.82 | 2.20 | 2.646(9) | 115 |
|  | O5-H5A...O1 | 0.82 | 2.31 | 2.973(10) | 138 |

**Table 2. Details of bond lengths (Å)**

| **Atoms**  **(A1-A2)** | **Bond lengths**  **Å** | **Atoms**  **(A1-A2)** | **Bond lengths,**  **Å** |
| --- | --- | --- | --- |
| O1—C1 | 1.350 (2) | O5—C17 | 1.359 (2) |
| O1—H1 | 0.8196 | O5—H5A | 0.8200 |
| O2—C2 | 1.354 (3) | O6—C22 | 1.364 (4) |
| O2—C15^i^ | 1.415 (5) | O6—C31 | 1.402 (3) |
| N1—C7 | 1.276 (4) | N3—C23^ii^ | 1.270 (3) |
| N1—N1^i^ | 1.399 (2) | N3—N3^ii^ | 1.406 (3) |
| C1—C6 | 1.367 (4) | C17—C18 | 1.355 (4) |
| C1—C2 | 1.396 (5) | C17—C22 | 1.389 (3) |
| C2—C3 | 1.372 (2) | C18—C19 | 1.382 (3) |
| C3—C4 | 1.391 (4) | C18—H18 | 0.9300 |
| C3—H3 | 0.9300 | C19—C20 | 1.376 (3) |
| C4—C5 | 1.378 (5) | C19—H19 | 0.9300 |
| C4—C7 | 1.447 (2) | C20—C21 | 1.387 (4) |
| C5—C6 | 1.382 (2) | C20—C23 | 1.451 (3) |
| C5—H5 | 0.9300 | C21—C22 | 1.366 (3) |
| C6—H6 | 0.9300 | C21—H21 | 0.9300 |
| C7—H7 | 0.9300 | C23—N3^ii^ | 1.270 (3) |
| C15—O2^i^ | 1.415 (5) | C23—H23 | 0.9300 |
| C15—H15A | 0.9600 | C31—H31A | 0.9600 |
| C15—H15B | 0.9600 | C31—H31B | 0.9600 |
| C15—H15C | 0.9600 | C31—H31C | 0.9600 |

Symmetry codes: (i) -*x*-1, -*y*, -*z*; (ii) -*x*, -*y*, -*z*.

**Table 3. Details of bond angles (°)**

| **Atoms A1-A2-A3** | **Bond Angles (°)** | **Atoms A1-A2-A3** | **Bond Angles (°)** |
| --- | --- | --- | --- |
| C1—O1—H1 | 109.3 | C17—O5—H5A | 109.6 |
| C2—O2—C15^i^ | 117.3 (2) | C22—O6—C31 | 117.2 (2) |
| C7—N1—N1^i^ | 113.1 (2) | C23^ii^—N3—N3^ii^ | 111.7 (3) |
| C6—C1—O1 | 119.1 (2) | O5—C17—C18 | 118.99 (18) |
| C6—C1—C2 | 119.7 (2) | O5—C17—C22 | 121.2 (2) |
| O1—C1—C2 | 121.16 (18) | C18—C17—C22 | 119.8 (2) |
| C3—C2—O2 | 125.5 (2) | C19—C18—C17 | 120.1 (2) |
| C3—C2—C1 | 120.07 (19) | C19—C18—H18 | 119.9 |
| O2—C2—C1 | 114.5 (2) | C17—C18—H18 | 119.9 |
| C2—C3—C4 | 120.1 (2) | C18—C19—C20 | 120.6 (2) |
| C2—C3—H3 | 119.9 | C18—C19—H19 | 119.7 |
| C4—C3—H3 | 119.9 | C20—C19—H19 | 119.7 |
| C3—C4—C5 | 119.3 (2) | C19—C20—C21 | 118.9 (2) |
| C3—C4—C7 | 121.1 (2) | C19—C20—C23 | 119.5 (2) |
| C5—C4—C7 | 119.53 (19) | C21—C20—C23 | 121.5 (2) |
| C6—C5—C4 | 120.56 (19) | C22—C21—C20 | 120.26 (19) |
| C6—C5—H5 | 119.7 | C22—C21—H21 | 119.9 |
| C4—C5—H5 | 119.7 | C20—C21—H21 | 119.9 |
| C5—C6—C1 | 120.2 (2) | O6—C22—C17 | 113.9 (2) |
| C5—C6—H6 | 119.9 | O6—C22—C21 | 125.93 (18) |
| C1—C6—H6 | 119.9 | C17—C22—C21 | 120.2 (2) |
| N1—C7—C4 | 122.16 (18) | N3^ii^—C23—C20 | 122.6 (2) |
| N1—C7—H7 | 118.9 | N3^ii^—C23—H23 | 118.7 |
| C4—C7—H7 | 118.9 | C20—C23—H23 | 118.7 |
| O2^i^—C15—H15A | 109.5 | O6—C31—H31A | 109.5 |
| O2^i^—C15—H15B | 109.5 | O6—C31—H31B | 109.5 |
| H15A—C15—H15B | 109.5 | H31A—C31—H31B | 109.5 |
| O2^i^—C15—H15C | 109.5 | O6—C31—H31C | 109.5 |
| H15A—C15—H15C | 109.5 | H31A—C31—H31C | 109.5 |
| H15B—C15—H15C | 109.5 | H31B—C31—H31C | 109.5 |

Symmetry codes: (i) -*x*-1, -*y*, -*z*; (ii) -*x*, -*y*, -*z*.


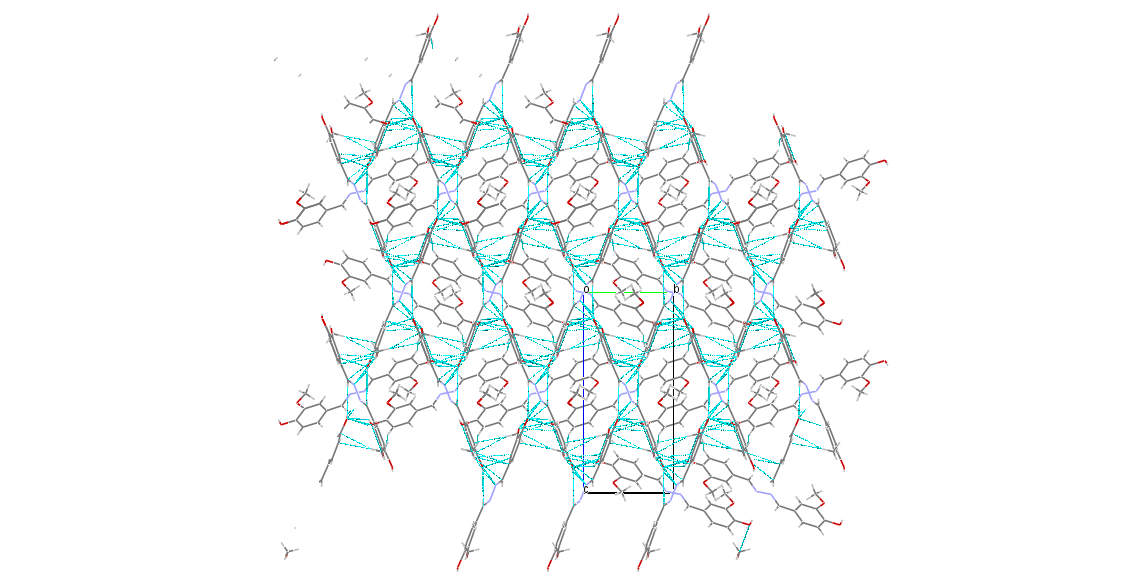


Figure 1. Various non-covalent interaction in azine along with ‘a’ axis
